# Robotic-Assisted Lobectomy in Non-Intubated Patient for Early Stage Non-Small-Cell Lung Cancer Treatment

**DOI:** 10.1093/icvts/ivag181

**Published:** 2026-06-29

**Authors:** Alessio Mariolo, Antoine Molina-Barragan, Maxime Giwerc, Eric Kovacs

**Affiliations:** Thoracic Surgery Department, Institut Montsouris, Curie-Montsouris Thoracic Institute, 750014 Paris, France; Department of Anesthesia and Intensive Care, Institut Montsouris, 75014 Paris, France; Department of Anesthesia and Intensive Care, Institut Montsouris, 75014 Paris, France; Thoracic Surgery Department, Institut Montsouris, Curie-Montsouris Thoracic Institute, 750014 Paris, France

**Keywords:** robotic-surgery, RATS, lung cancer, minimally invasive surgery, NITS, non-intubated thoracic surgery, innovation

## Abstract

Minimally invasive surgery is the gold standard for performing pulmonary lobectomy for early-stage non-small cell lung cancer. Recently, non-intubated video-assisted thoracoscopic surgery (NI-VATS), based on spontaneous ventilation under sedation and regional anaesthesia, has emerged as a less invasive alternative to conventional anaesthesia, reporting several benefits, but its application in major pulmonary resection remains controversial. We present the first report of robotic-assisted surgery (RATS) to perform a right lower lobectomy with radical lymph node dissection in a non-intubated patient presenting with pulmonary adenocarcinoma. In our experience, this approach is feasible, combining the ergonomic and precision advantages of robotic technology with the potential physiological benefits of spontaneous ventilation, furtherreducing the invasiveness of both surgery and anaesthesia. This innovative combination may be defined as non-intubated robotic-assisted thoracic surgery: NI-RATS.

## CASE REPORT

**Figure 1.**
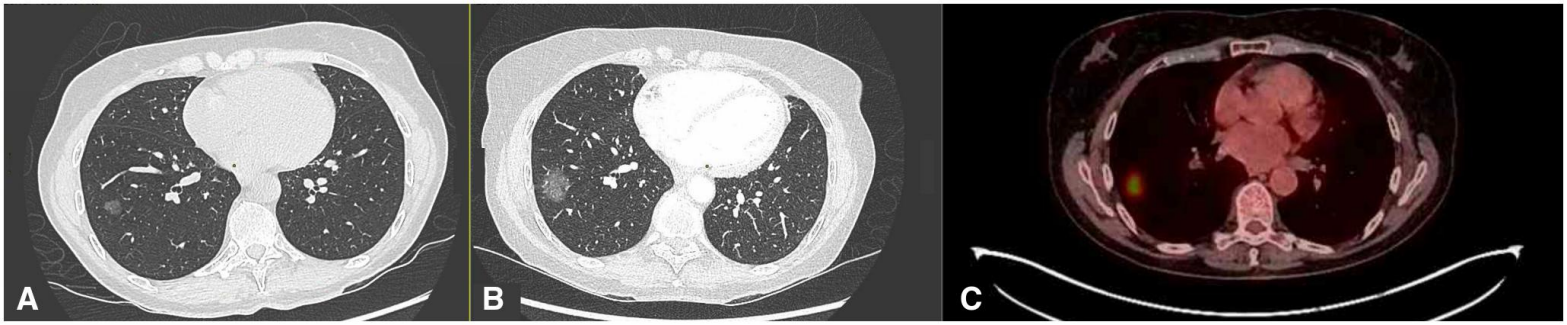
(A) Baseline CT-Scan (B) Preoperative CT-Scan 2 (C) Preoperative TEP-Scan.

A 62-year-old nonsmoker woman with an unremarkable medical history, was referred for a ground-glass opacity (GGO) lesion in the right lower lobe measuring 27 mm in diameter detected on a computed tomography scan (CT scan) conducted for persistent cough. Retrospective analysis showed that the lesion had already been present 8 years before, measuring 13 mm (**Figure 1A and B**). The patient was classified as ASA class II and was in good clinical condition with no relevant comorbidities with a body mass index of 24.8 kg/m^2^. A positron emission tomography (PET) scan demonstrated isolated hypermetabolism (standardized uptake value of 4.1) (**Figure 1C**). Brain MRI was negative. Respiratory functional tests were correct with a forced expiratory volume in 1 s (FEV1) of 75% and a diffusing capacity for carbon monoxide (DLCO) of 78%. The lesion was staged as cT1cN0M0 and, following a tumour board discussion, the patient was a candidate for surgical resection. Due to the size and localization of the lesion, a right lower lobectomy with radical lymphadenectomy was planned. After a multidisciplinary evaluation involving thoracic surgeons, anaesthesiologists and pulmonologists, the patient was considered an optimal candidate for non-intubated thoracic surgery due to the favourable body habitus and easy airways (Mallampati grade I), absence of persistent cough, coagulopathy, or compromised cardiovascular reserves. Anaesthesia was induced using target-controlled infusion (TCI) of propofol (6 µg/mL) and remifentanil (80 µg; 1.3 µg/kg) to facilitate the insertion of a laryngeal mask. Controlled ventilation was transitioned to spontaneous breathing. A laryngeal mask airway (LMA) was maintained throughout the procedure. Spontaneous ventilation represented the default ventilatory strategy, while assisted ventilation with pressure support was applied when necessary to maintain adequate ventilation and gas exchange. Target-controlled infusion propofol was used to maintain a deep hypnotic state targeting a bispectral index (BIS) between 40 and 60. The patient was monitored with an electrocardiogram, a pulse oximeter, invasive arterial pressure, and BIS. Continuous capnography through the LMA was also used to monitor end-tidal CO_2_. After anaesthetic preparation, she was positioned in a left lateral decubitus position.

Ultrasound-guided paravertebral block was performed at the T6-T8 intercostal spaces using 30 mL of 0.375% ropivacaine for regional anaesthesia and an additional 2 mL of lidocaine 1% with epinephrine 0.0005% was injected at each incision site before trocar placement. A 4-arm robotic approach using the da Vinci Xi system (Intuitive Surgical) was performed according to our standard procedure for fissure-based lobectomy (**Video 1**). An 8-mm trocar was inserted in the eighth intercostal space on the mid-axillary line, creating an iatrogenic pneumothorax, thus allowing the introduction of a 30° robotic endoscope.

**Figure 2.**
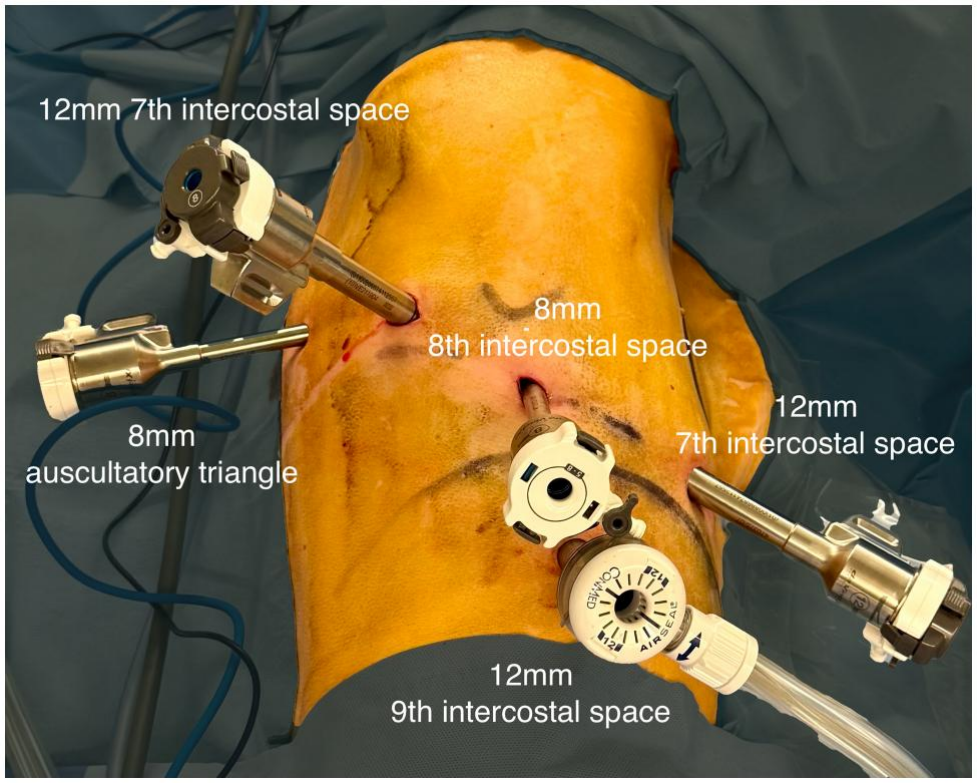
Trocars Position.

Opening of the pleural cavity produced an “open pneumothorax effect,” allowing partial collapse of the lung. An 8-mm port was placed in the auscultatory triangle, and two 12-mm ports in the seventh intercostal space along the anterior and posterior axillary lines, respectively. A 12-mm assistant trocar was inserted in the nineth intercostal space on the mid-axillary line allowing CO2 insufflation (pressure, 8 mmHg; flow, 20 L/min), which further facilitated lung deflation and surgical exposure (**Figure 2**). To minimize the cough reflex during lung and airway manipulation, a vagal nerve block was obtained by injecting a 5 mL of ropivacaine 1 mg/mL mixed with lidocaine 10 mg/mL into the paratracheal space. The triangular ligament was dissected, and the inferior pulmonary vein was isolated. A radical lymph node dissection of stations 8, 7, 2, and 4 was performed. Stations 10 and 11 were resected during hilar dissection. Once the fissure was opened, the superior segmental, common basal arteries, and inferior vein were cut using 45-mm EndoWrist vascular staplers. The lobar bronchus was divided using a 45-mm stapler, and the specimen was removed using an Endobag. No intraoperative complications occurred. No adverse events related to the absence of endotracheal intubation or double-lumen ventilation were observed, including cough, hypoxaemia, or hypotension. Arterial blood gases were obtained every 15 min: intraoperative PaCO_2_ remained within acceptable limits throughout the procedure (pH 7.15-7.34, CO_2_ 44.7-69.8 - mmHg) except during specimen extraction, when transient hypercapnia occurred (pH 7.15, CO_2_ 69.8 mmHg) due to hypoventilation following a sufentanil bolus administered before bronchial section. Assisted spontaneous ventilation (pressure support, 8-14 cmH_2_O) was initiated to mitigate hypercapnia and acidosis was rapidly corrected by increasing inspiratory pressure support. Surgery lasted 63 min, with a total blood loss of 10 mL. The patient was immediately awake in the operating room and reported no discomfort. A single pleural drain connected to a digital suction system was removed 4 hours after surgery. The postoperative course was uneventful, and the patient was discharged on postoperative day 1.

Postoperative analgesia consisted of our standard opioid-sparing regimen for minimally invasive thoracic surgery, based on the combination of oral paracetamol and ketoprofen. Early postoperative pain was well controlled, with a Numerical Rating Scale (NRS) score of 2/10 during the first postoperative day. Final pathological analysis showed a 25 × 18 mm invasive adenocarcinoma, with no vascular invasion or spread through air spaces (STAS) classed pT1cN0 R0 (stage IA3, nineth TNM edition) with a total of 24 resected lymph nodes. After a 20-day follow-up, the patient was in good physical condition without analgesic requirement.

## DISCUSSION

General anaesthesia with deep neuromuscular blockade and tracheal intubation using a double-lumen tube remains the standard approach for major anatomical pulmonary resections and is safely performed in the vast majority of thoracic procedures. However, general anaesthesia and mechanical ventilation are associated with adverse effects, including potential airway injuries, ventilator-induced lung damage, and postoperative nausea and vomiting.^1^ Recently, NI-VATS has emerged as a safe and feasible alternative for selected patients.^2^ This approach has demonstrated several advantages, including shorter operative time, reduced chest drain duration and hospital stay, attenuation of the inflammatory response, and potentially better long-term survival in patients operated on for cancer.^3^^,^^4^ Despite these, the adoption of NI-VATS for major anatomical resections remains controversial.^5^ The main concerns relate to the technical difficulty of precise exposition, dissection, and division of vascular and bronchial structures, as well as performing a complete lymphadenectomy under spontaneous ventilation, where respiratory and cough movements can interfere with the surgical field. RATS may overcome these limitations thanks to the enhanced 3D visualization, superior dexterity of the instruments, and the fixed trocar system reducing the impact of diaphragmatic excursion and chest wall motion during spontaneous ventilation. Nevertheless, this technique should be proposed to selected patients and performed in high-volume centres with established expertise in NITS and with a validated, simulation-tested safety protocol, including predefined conversion pathways and rehearsed management strategies for major intraoperative complications, that enables prompt conversion to endotracheal intubation when required (ie, persistent hypoxaemia, severe hypercapnia with respiratory acidosis, uncontrolled coughing, major bleeding, or inadequate surgical exposure).

To our knowledge, this is the first reported case of an NI-RATS lobectomy. The absence of intraoperative and anaesthetic complications supports the feasibility of this innovative approach. Our experience encourages further investigation of NI-RATS as a potential new frontier in minimally invasive pulmonary resection.

## DATA AVAIBILITY

The data underlying this article will be shared on reasonable request to the corresponding author.
